# Fibrinolytic therapy in patients with ST-segment elevation myocardial infarction: Accelerated versus standard Streptokinase infusion regimen

**DOI:** 10.15171/jcvtr.2017.36

**Published:** 2017-12-30

**Authors:** Ahmed Bendary, Wael Tawfik, Mohamed Mahrous, Mohamed Salem

**Affiliations:** Cardiology Department, Benha University Hospital, Benha Faculty of Medicine, Egypt

**Keywords:** Myocardial infarction, Streptokinase, Reperfusion

## Abstract

***Introduction:*** Timely fibrinolysis for acute ST-segment elevation myocardial infarction (STEMI) reduces infarct size and hence preserves LV function and reduces mortality. Optimal regimen of streptokinase (SK) infusion in such patients is a matter of interest. The current study aimed to compare efficacy and safety of accelerated SK infusion regimen in patients with STEMI versus the standard one.

***Methods:*** One hundred consecutive STEMI patients were randomly allocated into one of 2 groups: group I (50 patients) who received accelerated SK regimen (1.5 million units over 30 minutes) and group II (50 patients) received standard SK regimen (1.5 million units over 60 minutes). Efficacy was evaluated non-invasively using clinical (chest pain), ECG (resolution of ST segment) and laboratory tests (earlier and higher peaking of cardiac troponin I). Safety was evaluated by assessment of multiple in-hospital adverse events.

***Results:*** Both groups were statistically matched in all baseline criteria. There was a significant difference between both groups regarding each parameter of successful reperfusion in favor of accelerated regimen. When all these parameters were combined, 31 patients (62%) had successful reperfusion in group I versus 19 patients (38%) in group II (*P *= 0.016). We did not report any significant difference between both groups regarding in-hospital mortality, in-hospital heart failure, major bleeding, hypotension or allergic reaction to SK. Mean pre-discharge ejection fraction was higher in group I than group II (50.9 ± 6.6% versus 47.3 ± 4.6%, *P *= 0.002).

***Conclusion:*** Accelerated regimen of SK infusion is safe and effective method of reperfusion in patients with STEMI.

## Introduction

The pathophysiology of ST-segment elevation myocardial infarction (STEMI) entails acute vulnerable plaque change that includes: plaque rupture, fissuring or erosion, all of which could result in total arterial occlusion if stimulus for thrombus formation is potent and collateral circulation is not well developed.^1^ Since morbidity and mortality due to myocardial infarction closely correlate with its size, timely reperfusion of the infarct-related artery (either by fibrinolysis or primary percutaneous coronary intervention [PCI]) is the mainstay of therapy to preserve left ventricular function (the main predictor of survival in such cases).^2^ Streptokinase (SK) is the most widely used fibrinolytic agent especially in economically burdened countries due to the higher cost of the more effective recent generations of fibrinolytics such as tissue plasminogen activator (t-PA).^3^ Most randomized trials used a slow infusion of SK over 60 minutes, this may have been due to concerns regarding ensuing hypotension or hemorrhagic complications of SK with faster regimens. However, some evidence points to the fact that a minimum dose of 500 U/kg/min may be needed for effective tissue level reperfusion, a dose that can not be obtained with such a relatively slow infusion over 60 minutes.^4^

The objective of this study was to compare efficacy and safety of a new faster SK infusion versus old traditional regimen in patients with STEMI.

## Materials and Methods

### Study design

This prospective randomized single-blinded, single-center study included 100 consecutive patients with STEMI who were admitted to the coronary care unit (CCU) at Benha University Hospital, Egypt in the period from December 2016 to June 2017. We aimed to test safety and efficacy of accelerated SK regimen as compared to the standard one. *Key inclusion criteria* were: Age between 18-75 years, of either sex, chest pain typical for myocardial infarction of at least 30 minutes duration and less than 6 hours, new ST segment elevation in two contiguous leads with the cut-off points ≥0.2 mV in men or ≥0.15 mV in women in leads V2-V3 and/or ≥0.1 mV in other leads on the 12–lead ECG. All patients were candidates for reperfusion therapy by immediate fibrinolysis if timely PPCI was confirmed to be unavailable for whatever reason, with possible rescue PCI if fibrinolysis was found to be unsuccessful. While *Key exclusion criteria* were: Absolute or relative contraindications to SK, including prior intracranial hemorrhage, structural cerebral vascular lesion, ischemic stroke within 3 months, suspected aortic dissection, active bleeding or bleeding diathesis, significant closed head or facial trauma within 3 months, severe poorly controlled hypertension.

### Baseline and in-hospital evaluation

All patients had review of medical history, full general and cardiac examination, evaluation of chest pain every 10 minutes during the first 90 minutes after starting SK infusion to detect the onset of clinical reperfusion and chest pain was described as ongoing or absent. Continuous ECG monitoring using the lead that showed highest ST-segment elevation to detect possible arrhythmias. Standard 12-lead ECG was recorded at the presentation to the emergency room (ER), 90 minutes after fibrinolytic therapy and every 24 hours during the in-hospital stay. Serial measurements of plasma levels of cardiac troponin I (cT-I) on admission, 12 hours and 24 hours later was done to detect peak levels.

#### Criteria for successful reperfusion

1. Disappearance of chest pain within 90 minutes of starting SK infusion.
2. Resolution of ST-segment elevation by more than 50% after starting SK infusion in the lead with maximum elevation on baseline ECG.
3. Earlier and higher peak of cardiac troponin I within the first 24 hours after onset of symptoms.
4. May be the appearance of reperfusion arrhythmias e.g. accelerated idioventricular rhythm (AIVR).

### Study protocol

All patients were randomly allocated using simple 1:1 randomization based on the given SK regimen, into 2 groups:

Group (I): Accelerated regimen (1.5 million units over 30 minutes)
Group (II): Standard regimen (1.5 million units over 60 minutes)

All patients received the same type of SK (Sedonase^®^, SEDICO pharmaceutical company, October city, Egypt), aspirin (300 mg), clopidogrel (300 mg) loading dose followed by maintenance dose of 75 mg daily, LMWH (1 mg/kg every 12 hours) during the stay in CCU. In addition, nitrates, angiotensin-converting enzyme inhibitors, ß-blockers, statins were given.

### Study end-points

In-hospital efficacy and safety of the accelerated regimen of SK versus standard regimen, including rate of successful reperfusion, mortality, major bleeding, symptomatic hypotension (defined as blood pressure (BP) <90/60 mm Hg measured non-invasively) and allergic complications.

### Statistical analysis

Data management and statistical analysis were performed using SPSS software version 23. Numerical data were summarized using means and standard deviations. Categorical data were summarized as numbers and percentages. Comparison between two groups with respect to the normally distributed numeric variable was done using independent *t* test. For categorical variables, differences were analyzed with chi-square test. Stepwise logistic regression was done to give adjusted odds ratio and measure magnitude of the effect of different factors on successful reperfusion. All *P* values were two-sided. *P* values <0.05 will be considered significant.

## Results

### Study population

The mean age was 56 ± 11 years (57 ± 11, 56 ± 11 years in group I and II respectively, *P* = 0.8). Eighty percent were males, 45% had a history of DM, 28% were hypertensives, 59% were smokers, 7% were obese, 18% were dyslipidemic, 13% had a family history of premature CAD, 24% had past history of diagnosed CAD and 15% had prior coronary interventions. Between groups analysis did not reveal statistically significant difference in baseline characteristics (Table 1).

**Table 1:** Baseline characteristics of study population

	**Group I, n=50**	**Group II n=50**	***P***
Age (y), mean ±SD	57 ± 11	56 ±11	0.804
Sex, male, No. (%)	40 (80)	40 (80)	1.0
DM, No. (%)	20 (40)	25 (50)	0.315
HTN, No. (%)	16 (32)	12 (24)	0.373
Smoking, No. (%)	28 (56)	31 (62)	0.542
Obesity, No. (%)	4 (8)	3 (6)	1.0
Dyslipidemia, No. (%)	8 (16)	10 (20)	0.603
Family history of premature CAD, No. (%)	8 (16)	5 (10)	0.372
Past history of CAD, No. (%)	12 (24)	12 (24)	1.0
Prior coronary intervention, No. (%)	7 (14)	8 (16)	0.779

Abbreviations: DM, diabetes mellitus; HTN, hypertension; CAD, coronary artery dsease.

### Total ischemic time

**Figure 1:**
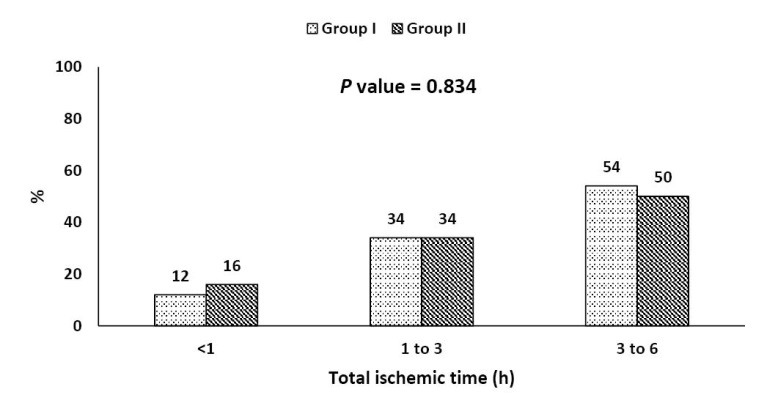
Total ischemic time in both groups.

Fibrinolysis was started within 1 hour from symptom onset in 12% of patients in group I versus 16% in group II. Total ischemic time was 1 to 3 hours in 34% in both groups and it was from 3 to 6 hours in 54% in group I versus 50% in group II. There was no statistically significant difference between both groups regarding all categories of total ischemic time (*P* = 0.83 for all) (Figure 1).

### Target infarction site as suggested by ECG

**Figure 2:**
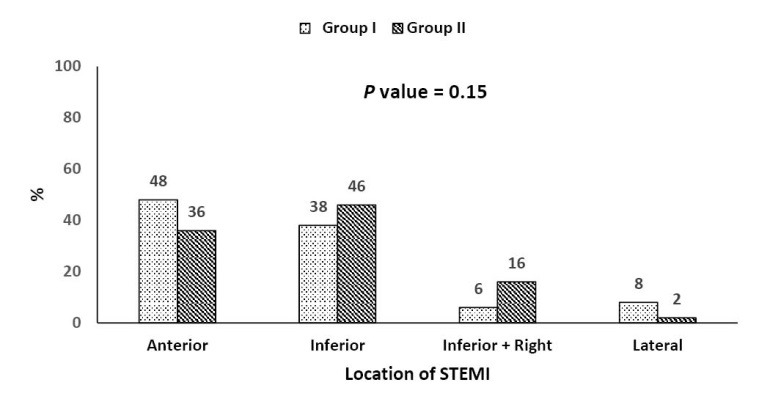
Target infarction site as suggested by ECG.

Anterior STEMI was reported in 42% of all patients (48%, 36% in group I and II respectively). Inferior STEMI was reported in 42% of all patients (38%, 46% in group I and II respectively). Inferior STEMI with right ventricular (RV) involvement was reported in 11% of all patients (6%, 16% in group I and II respectively). Lateral STEMI was reported in 5% of all patients (8%, 2% in group I and II respectively) (*P* = 0.15 for all; Figure 2).

### Efficacy parameters (successful reperfusion)

**Figure 3:**
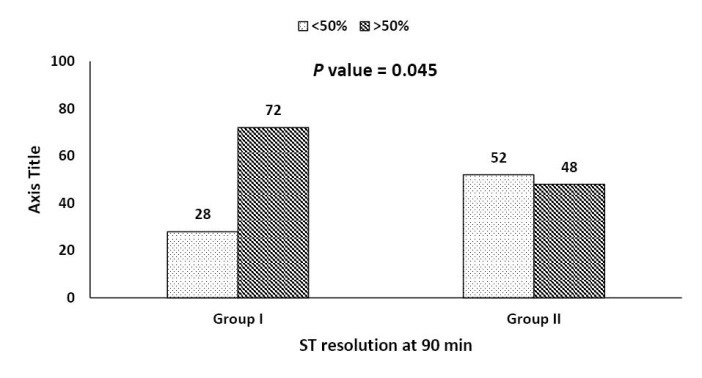
ST segment resolution after fibrinolysis.

**Figure 4:**
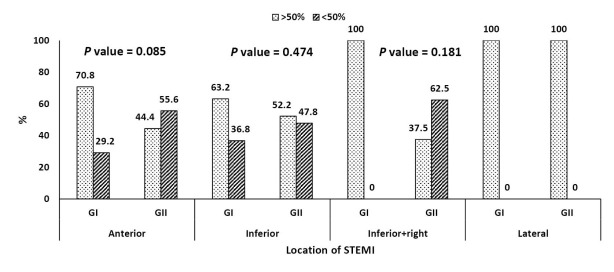
ST-segment resolution (stratified by location of STEMI).

ST-segment resolution by more than 50% was achieved in 60% of all patients (72%, 48% in group I and II respectively, *P* = 0.045). Less than 50% resolution of ST segment was reported in 40% of all patients (28%, 52% in group I and II respectively, *P* = 0.045) (Figure 3). This was found to be consistent when stratified by location of STEMI (Figure 4). Chest pain disappeared within 90 minutes of SK infusion in 32 patients in group I (64%) versus 21 patients in group II (42%) (*P* = 0.028). The percent of patients who achieved early peaking (less than 12 hours) of cardiac Troponin I was 68% in group I versus 46% in group II (*P* = 0.03). AIVR was the predominant reperfusion arrhythmia noted, it was observed in 8 patients in group I (16%) versus 11 patients in group II (22%) (*P* = 0.44).

When all the above-mentioned parameters for successful reperfusion were found, successful reperfusion was reported in 31 patients in group I (61%) versus 19 patients in group II (38%) (*P* = 0.016).

### Predictors of successful reperfusion

All patients were classified as those with successful reperfusion (50 patients, 50%) and those without successful reperfusion (50 patients, 50%) according to the previous predefined criteria. Logistic regression model was done to find out significant independent predictors for successful reperfusion using successful reperfusion as a dependent factor while demographic data, risk factors, total ischemic time, the location of STEMI and regimen of SK infusion were used as independent factors. It was found that significant independent predictors for successful reperfusion are: Accelerated regimen of SK (odds ratio [OR] = 3.77, 95% CI: 1.5-10, *P* = 0.006), total ischemic time <1 hour when compared to 3-6 hours (OR = 11.4, 95% CI: 2.5-51.5, *P* = 0.001), total ischemic time 1-3 hours when compared to 3-6 hours (OR= 5.7, 95% CI: 2.1-15.6, *P* = 0.001). Of note, demographic data, risk factors and location of STEMI as suggested by ECG did not correlate significantly with rates of successful reperfusion.

### In-Hospital outcome

In-Hospital mortality was reported in 3 patients (3%). Eighteen patients (18%) developed in-hospital heart failure. Major bleeding requiring blood transfusion was reported in 3 patients (3%) in the form of 2 patients with hematuria and 1 patient with hematemesis. Twenty-five patients (25%) developed SK induced symptomatic hypotension. Twenty-three patients (23%) underwent rescue PCI after failure of reperfusion by SK. Only 3 cases (3%) developed allergic reaction to SK.

Between groups analysis showed no statistically significant difference between both regimens in the adverse events nor in rates of rescue PCI (numerically higher in group II). Importantly, the mean LVEF was significantly higher in group I when compared to group II (Table 2).

**Table 2:** In-hospital outcome in both groups

	**Group I,** **n=50**	**Group II,** **n=50**	***P***
EF (%), mean ±SD	50.9 ±6.6	47.3 ±4.6	0.002
Hypotension, No. (%)	10 (20)	15 (30)	0.248
Major bleeding, No. (%)	1 (2)	2 (4)	1
Allergy, No. (%)	2 (4)	1 (2)	1
In-hospital heart failure, No. (%)	9 (18)	9 (18)	1
In-hospital mortality, No. (%)	1 (2)	2 (4)	1
Rescue PCI, No. (%)	8 (16)	15 (30.0)	0.096

Abbreviations‏: EF, Ejection fraction, PCI, Percutaneous coronary intervention.

## Discussion

Fibrinolytic therapy for reperfusion of the infarct-related artery is one of the most important developments in the management of STEMI during last 45 years. Fibrinolytic therapy when combined with Aspirin and Heparin resulted in significantly higher rates of reperfusion and reduction of infarct size (the major determinant of LV function), and this has been translated into significantly lower mortality compared to the conservative treatment of this condition.^5^ For understandable reasons related to the potential risk of hypotension (a well known specific effect of SK) and hemorrhagic complications, standard SK regimen (1.5 million units over 60 minutes) remained unchanged during last 15 years.^4^

In this prospective randomized clinical study, we found that accelerated regimen of SK is safe and well-tolerated (no significant increase in major bleeding, hypotension or allergy) and more effective (significantly higher rates of successful coronary reperfusion) compared to the standard one.

The minimum required dose of SK for effective coronary reperfusion has been measured to be around 500 IU/kg/min.^6^ Therefore, an average 70 kg patient will need a minimum dose of 35000 units/min for reperfusion. If this patient was given the conventional regimen of 1.5 million units/60 min, he/she will only receive 25000 units/min of SK compared to 75000 units/min in the accelerated regimen.

As compared to lower serum concentrations, higher concentrations of SK result in a larger number of SK-plasminogen/plasmin complexes, decreasing the availability and hence, systemic activity of free plasmin which may result in reduction of hemorrhagic complications. However, SK complexes with plasminogen/plasmin and free SK may still have the ability to activate plasminogen in clots, inducing intense local fibrinolysis.^7^ Therefore, it has been shown that the major determinant of clot dissolution is its components and not the fibrinolytic agent itself, provided that the serum concentration of the fibrinolytic agent remains sufficient.^8^ This strengthens our hypothesis that a higher concentration of SK or SK-plasminogen/plasmin complexes will result in a higher extent of penetration of the agent into the clot and thus the degree of successful tissue level reperfusion.^9^

The results of the present study reaffirm the above-mentioned theories and are in accordance with the results of previous studies showing that standard regimen for SK infusion used in major trials may not be the optimal one.

In this study, 62% of patients receiving accelerated regimen compared to 38% of those receiving the traditional regimen had successful reperfusion assessed non-invasively (*P* = 0.016).

In one recent study,^10^ 74% of patients receiving accelerated regimen compared to 48% of patients receiving the standard regimen had successful reperfusion. Moreover, in a multivariate model, accelerated regimen was found to be the most important predictor for successful reperfusion (OR = 3.5, 95% CI: 1.5-6.3, *P* = 0.01).

Ghaffari et al^11^ performed a randomized double-blind clinical trial comparing an accelerated infusion (1.5 MU/20 min) with the conventional infusion (1.5 MU/60 min) of SK in 300 patients with their first episode of acute STEMI. In multivariate analysis, accelerated SK infusion was the only independent predictor of higher coronary reperfusion (OR = 3.2, CI: 1.93-5.3, *P* < 0.001).

In the ASK-ROMANIA multicenter registry,^12^ 1880 consecutive patients admitted within 6h of STEMI onset were allocated one of the following 4 SK regimens: 1.5 M.U. over 60 minutes, 1.5 M.U./30 min, 1.5 M.U./20 min, 0.75 M.U./10 min, repeated or not after 50 minutes if no electrocardiographic criteria of reperfusion. It was found that rates of coronary reperfusion (non-invasively detected) for SK1.5/30 (72.39%), SK1.5/20 (75.34%) and SK0.75/10 (72.85%) were similar but higher than for SK1.5/60 (64.03%).

The ASENOX study^13^ included 633 consecutive patients, admitted within 6 hours after the onset of STEMI. The rates of coronary reperfusion in the accelerated SK plus enoxaparin (77.6%) and accelerated SK plus UFH (73.5%) groups were similar but both were significantly higher than that observed in the standard SK plus UFH group (62.2%) (*P* = 0.002 and 0.013, respectively).

The present study showed lower in-hospital mortality among patients who received accelerated SK regimen (2%) compared to those who received traditional regimen (4%) although it did not reach statistical significance. These results are in agreement with ASENOX study,^13^ which reported 6.06% mortality rates with accelerated SK in STEMI patients compared to 12.74% in the standard regimen.

In our study, there was no significant difference between both groups regarding the rate of in-hospital major bleeding, symptomatic hypotension and allergic reactions to SK, a similar finding to those reported in prior studies.^10-13^ This confirms that accelerated regimen of SK is, at least, as safe as the standard one. Noteworthy, symptomatic hypotension was numerically higher in group II than group I but it did not reach statistical significance. This may be explained by small sample size.

Mean pre-discharge EF was significantly higher in patients receiving accelerated SK regimen when compared to those who received traditional regimen (50.9 ± 6.6% versus 47.3 ± 4.6% respectively, *P* = 0.002), a result that is closely similar to that reported by Ghaffari et al.^11^

## Conclusion

Accelerated regimen of SK infusion is safe, effective, and well-tolerated in patients with acute STEMI.

## Study limitations

- Small number of patients (only 100). A larger sample size could yield more significant results.
- Success of reperfusion was assessed only non-invasively.
- Patients were followed up only for a short term (during in-hospital stay).

## Competing interests

All authors disclose no conflicts of interest.

## Ethical approval

Our institutional review board and ethics committee approved the performance of this research, and all patients signed a written informed consent.

## Acknowledgments

Authors would like to thank all staff of Coronary Care Unit at Benha University Hospital for their support.
